# Comparison of therapeutic efficacy and toxicity of docetaxel, cisplatin, and fluorouracil (TPF)-based induction chemotherapy plus concurrent chemoradiotherapy and chemoradiotherapy alone in locally advanced nasopharyngeal carcinoma

**DOI:** 10.1097/MD.0000000000027475

**Published:** 2021-10-22

**Authors:** Ruijuan Chen, Yongkai Lu, Yuemei Zhang, Ruixin He, Fengwen Tang, Wei Yuan, Yi Li, Xiaowei Zhang

**Affiliations:** a Department of Xi’an Central Hospital, the Affiliated Hospital of Xi’an Jiaotong University, Xi’an, Shaanxi, China; b Department of Radiotherapy, Oncology Department, the First Affiliated Hospital of Xi’an Jiaotong University, Xi’an, Shaanxi Province, China.

**Keywords:** cisplatin, concurrent chemoradiotherapy, docetaxel, fluorouracil, meta-analysis, nasopharyngeal carcinoma

## Abstract

**Purpose:**

In recent years, docetaxel, cisplatin, and fluorouracil (TPF)-based induction chemotherapy plus concurrent chemoradiotherapy (CCRT) has been commonly applied for locally advanced nasopharyngeal carcinoma (LA-NPC). However, whether TPF+CCRT regimen is the best choice for LA-NPC remains unclear. This meta-analysis aims to elucidate and compare the efficacy and toxicity of TPF+CCRT versus CCRT alone for LA-NPC.

**Methods:**

Two investigators independently and systematically searched relevant studies available on PubMed, Embase, Cochrane Library, and Web of Science published before January 7, 2021. Data were extracted from eligible studies for assessing their qualities, and calculating pooled hazard ratios (HR), odds ratio (OR) and 95% confidence intervals (CI) using Review Manager software 5.3 (RevMan 5.3).

**Results:**

Five studies involving 759 LA-NPC patients were analyzed in the meta-analysis. Compared to CCRT alone, TPF-based IC plus CCRT significantly improved overall survival (OS) (HR = 0.53, 95% CI: 0.35–0.81, *P* = .003), progression-free survival (PFS) (HR = 0.63, 95% CI: 0.46–0.86, *P* = .004), distant metastasis-free survival (DMFS) (HR = 0.58, 95% CI: 0.39–0.86, *P* = .008), and locoregional failure-free survival (LRFFS) (HR 0.62, 95% CI: 0.43–0.90, *P* = .01). In addition, TPF-based IC plus CCRT mainly increased risks of grade 3/4 acute hematological toxicity and non-hematological toxicities like leukopenia (OR = 1.84, 95% CI: 0.42–8.03, *P* = .42), neutropenia (OR = 1.78, 95% CI: 0.23–13.82, *P* = .58), thrombocytopenia (OR = 1.76, 95% CI: 0.53–5.81, *P* = .35), febrile neutropenia (OR = 2.76, 95% CI: 0.07–101.89, *P* = .58), vomiting (OR = 18.94, 95% CI: 0.99–362.02, *P* = .05) and dry mouth (OR = 2.23, 95% CI: 0.22–22.57, *P* = .50), which were uncomplicated and manageable.

**Conclusions:**

TPF + CCRT is superb than CCRT alone for the management of LA-NPC. However, TPF+CCRT increases the incidences of grade 3/4 acute hematological toxicity and some non-hematological toxicities.

## 1 Introduction

Cancers of the pharynx (nasopharynx, oropharynx, and hypopharynx) together accounted for 302,000 new cancer cases worldwide estimated in 2018, of which about 40% were nasopharyngeal carcinoma (NPC).^[1]^ NPC is an epithelial malignancy with endemic and racial distributions, and it has an extremely high prevalence in Southeast Asia, North Africa, and Southern China.^[2]^ Due to its hidden anatomical location and atypical symptoms, approximately 70% to 80% of NPC cases are diagnosed at locally advanced (LA) stage.^[3]^ Concurrent chemoradiotherapy (CCRT) is one of the standard treatments for locally advanced nasopharyngeal carcinoma (LA-NPC).^[4–6]^ At present, the well-known clinical application of CCRT is mainly supported by two-dimensional radiotherapy,^[7]^ clinical evidences of it in the field of intensity-modulated radiotherapy are lacked.^[8]^ In addition, distant metastasis is still the predominant cause of treatment failure, and about 20% to 30% of LA-NPC patients develop distant metastases after CCRT.^[9,10]^

Induction chemotherapy plus concurrent chemoradiotherapy (IC+CCRT) has gradually been shown superior to CCRT in the management of LA-NPC, manifesting as higher overall survival (OS), progression-free survival (PFS) and distant metastasis-free survival (DMFS).^[11–14]^ Thus, IC+CCRT, a promising treatment strategy, is recommended by latest National Comprehensive Cancer Network guidelines.^[15]^ Induction regimens, including PF (cisplatin and 5-fluorouracil), docetaxel and cisplatin, and TPF (docetaxel, cisplatin, and 5-fluorouracil), are usually applied in chemotherapy of LA-NPC.^[7]^ However, the optimal IC regimen has not been established.

The most effective IC regimen of LA-NPC, at present, is unclear, and the conclusion remains inconsistent. Aiming to provide direct and indirect evidences for the final selection of the IC regimen, we carried out a meta-analysis to compare the toxicity, safety, and efficacy of the TPF + CCRT and the CCRT alone in LA-NPC patients.

## 2 Methods

### 2.1 Search strategy

Using a combination of medical subject heading terms and/or free text words as follows, we thoroughly searched relevant studies published before January 7, 2021 in 4 medical databases including Pubmed, Embase, Cochrane library, and Web of science: “nasopharyngeal carcinoma,” “induction chemotherapy,” “chemoradiotherapy,” “docetaxel,” “cisplatin,” and “fluorouracil”. There was no limitation on the language of published studies. Furthermore, references of selected studies were manually reviewed. Literature search and screen were independently performed by 2 investigators. Disagreement was resolved by discussion with a third investigator.

### 2.2 Inclusion criteria

All included studies were in line with the principles of Participants, Intervention, Comparison, and Outcomes, Study design. Inclusion criteria were as follows:

1. Participants [P]: Patients were pathologically diagnosed with locoregional advanced NPC without distant metastasis.
2. Intervention [I]: Patients in the experimental group received TPF plus CCRT.
3. Comparison [C]: CCRT alone was the intervention in control group.
4. Outcomes [O]: The outcomes included overall survival (OS), progression-free survival (PFS), DMFS, local failure-free survival (LRFFS) and related adverse events;
5. Study design [S]: Randomized controlled trials (RCTs) and observational studies, including cohort and case-control studies.

### 2.3 Exclusion criteria

Articles satisfying any of the following items were excluded:

1. Reviews, case reports, letters, abstracts;
2. Low research quality or high-risk of bias;
3. Available data that could be pooled were lacked.

### 2.4 Data extraction

The following information were independently extracted from the included studies by 2 researchers (Ms. Zhang and Ms. He): First author, year of publication, country, study design, age, histological type, clinical tumour stage, primary endpoint, sample size, follow-up duration, detailed treatment plan and outcomes of the various subgroups. A dispute regarding data extraction was intervened by the third investigator (Mr. Tang).

### 2.5 Quality assessment

Two evaluation scales were used in this study, including the Cochrane risk of bias tool and Newcastle-Ottawa Scale. The former one was used for RCTs, involving 7 items:

1. Random sequence generation;
2. Allocation concealment;
3. Blinding of participants and personnel;
4. Blinding of the outcome assessment;
5. Incomplete outcome data;
6. Selective reporting, and
7. other bias. Each item was assessed as having a high, low or unclear risk of bias.^[16]^

Newcastle-Ottawa Scale was introduced to assess the risk of bias in non-RCTs, involving 3 perspectives: Selection, comparability and outcome of studies.^[16]^ It was a 0 to 9 scale, in which 4 points were graded for selection, 2 for comparability and 3 for outcomes. Studies with 6 points or higher were considered as high quality.^[17–19]^

### 2.6 Statistical analysis

The pooled statistics were performed using RevMan software version 5.3 (Cochrane Collaboration, Oxford, UK). The hazard ratio (HR) was selected as the effect indicator to synthesize time-to-event endpoints (OS, PFS, DMFS, and LRFFS) based on the methodology published by Tierney et al.^[20]^ Engauge Digitizer software was used to extract the HR from the survival curve when the HR was not directly described in the included articles. The incidence of adverse events was calculated through odds ratio (OR) to assess the strength of the association. Heterogeneity between trials was evaluated through the Cochrane Q test and the *I*^2^ statistic, which quantified the proportion of total variation caused by heterogeneity instead of chance.^[16]^ If the *P* value of the *Q* test was >0.10 and *I*^2^ <50%, a fixed-effects model was used for data with nonsignificant heterogeneity; Otherwise, a random-effects model was used for data with significant heterogeneity.^[21,22]^ Furthermore, the sensitivity analysis was also applied to examine the potential influence of an individual study on the overall assessment by removing one study each time and pooling the remaining trials. Due to the limited number of included studies (<10), the Begg and Egger tests were not performed to assess the publication bias.^[23–25]^

## 3 Results

### 3.1 Study selection

**Figure 1:**
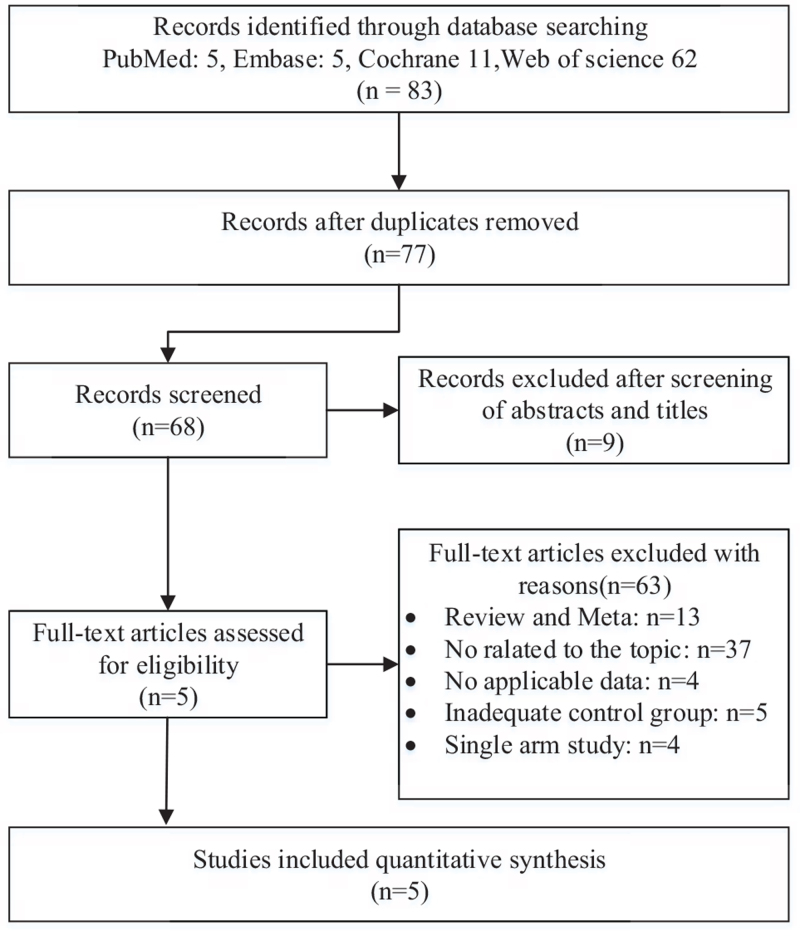
Flow chart of the search process for the meta-analysis.

Initially, 77 articles were retrieved through the preliminary search in PubMed, Embase, the Cochrane Library, and Web of science after excluding 6 duplicates. Then, 72 ineligible ones were eliminated through reviewing titles and abstracts. After full-text reading, 5 eligible articles were assessed for design and quality.^[26–30]^ The detailed process of study selection was shown in Figure 1.

### 3.2 Study characteristics

Finally, 5 studies^[26–30]^ with a total of 759 LA-NPC patients were included in our meta-analysis. 2/5^[27,29]^ were RCTs, and the remaining^[26,28,30]^ were retrospective studies. Furthermore, all included studies were identified as high quality by Cochrane Collaboration and the Newcastle–Ottawa Scale. The baseline information of the 5 included studies were summarized in Table 1.

**Table 1:** Characteristics of the studies included in the meta-analysis.

Included study, year, country	Inclusion period	Clinical stage	Study design	Study type	TPF-based induction chemotherapy	Concurrent chemoradiotherapy	Patients (TPF+CCRT/ CCRT alone)	Median follow-up (months)	Outcomes	NOS score
Ou et al,^[25]^ France	1999.01–2012.06	AJCC 7th(III-IVb)	TPF+CCRTCCRT	Re.	Docetaxel75 mg/m^2^ dL; cisplatin75 mg/m^2^ dL;fluorouracil750 mg/m^2^ dL–5; q3wks×3	Cisplatin100 mg/m^2^ dL, q3wks × 3; or cisplatin 40 mg/m^2^ dL, q1wk×7(maximum); or carboplatin (AUC2) d1, q1wk×7 (maximum).Radiotherapy:3D-CRT or IMRT.	58/48	76.8 (3.6–172.8)	OS, PFS,DMFS,LRFFS,acute toxicity	6
Sun et al,^[26]^ China	2011.03–2013.08	UICC/AJCC 7th edition III-IV(except T3-4N0)	TPF+CCRTCCRT	RCT	Docetaxel60 mg/m^2^ dL; cisplatin60 mg/m^2^ dL;fluorouracil600 mg/m^2^ dL-5; q3wks × 3	Cisplatin100 mg/m^2^d1 q3wks × 3;Radiotherapy: IMRT	241/239	45 (38-49)	OS, PFS,DMFS,LRFFS,acute toxicity	NA
Kawahira et al,^[27]^ Japan	2006.10–2016.05	N2-N3(III–IVB))	TPF+CCRTCCRT	Re.	Docetaxel60–70 mg/m^2^ dL; cisplatin60-70 mg/m^2^ dL;5-fluorouracil750–800 mg/m^2^ dL–5;q3wks × 3	Cisplatin (q3wks; 80–100 mg/m^2^ on dL, 22, and 43), split cisplatin (q3wks 20 mg/m^2^ on dL–4, 22–25,43-46), or cisplatin (q1wk; 40 mg/m^2^ on dL, 8, 15, 22, 29, 36, 42), and carboplatin (AUC2) on dL, 8, 15, 22, 29, 36,42 at the physicians’ discretion.Radiotherapy:3D-CRT or IMRT	12/16	TPF+CCRT:36.4 (6.7–55.2)CCRT: 40.1 (4.3–99.0)	OS, PFS,DMFS,LRFFS,acute toxicity	8
Included study, year, country	Inclusion period	Clinical stage	Study design	Study type	TPF-based induction chemotherapy	Concurrent chemoradiotherapy	Patients (TPF+CCRT/ CCRT alone)	Median follow-up (months)	Outcomes	NOS score
Frikha et al,^[28]^2018, France and Tunisia	2009-2012	T2b, T3, T4 and/or N1-N3, M0	TPF+CCRTCCRT	RCT	Docetaxel75 mg/m^2^ dL; cisplatin75 mg/m^2^ dL;fluorouracil750 mg/m^2^ dL-5; q3wks × 3	Cisplatin 40 mg/m^2^; d1, weekly;radical radiotherapy	42/41	43.1 (42.3–45.0)	OS, PFS,DMFS,LRFFS,acute toxicity	NA
Mnejja et al,^[29]^ 2018, Tunisia	2004.06–2009.01	T1, T2a, T2b,T3, T4 and/or N0-N3b and/or AJCC 6th (IIb-IVb)	TPF+CCRTCCRT	Re.	Docetaxel75 mg/m^2^ dL; cisplatin75 mg/m^2^ dL;fluorouracil750 mg/m^2^ d1-5; q3wks × 3	Cisplatin 40 mg/m^2^; dL, weekly;radical radiotherapy	32/30	53.5 (40–87)	OS, PFS,DMFS,LRFFS,acute toxicity	6

### 3.3 Overall survival

**Figure 2:**
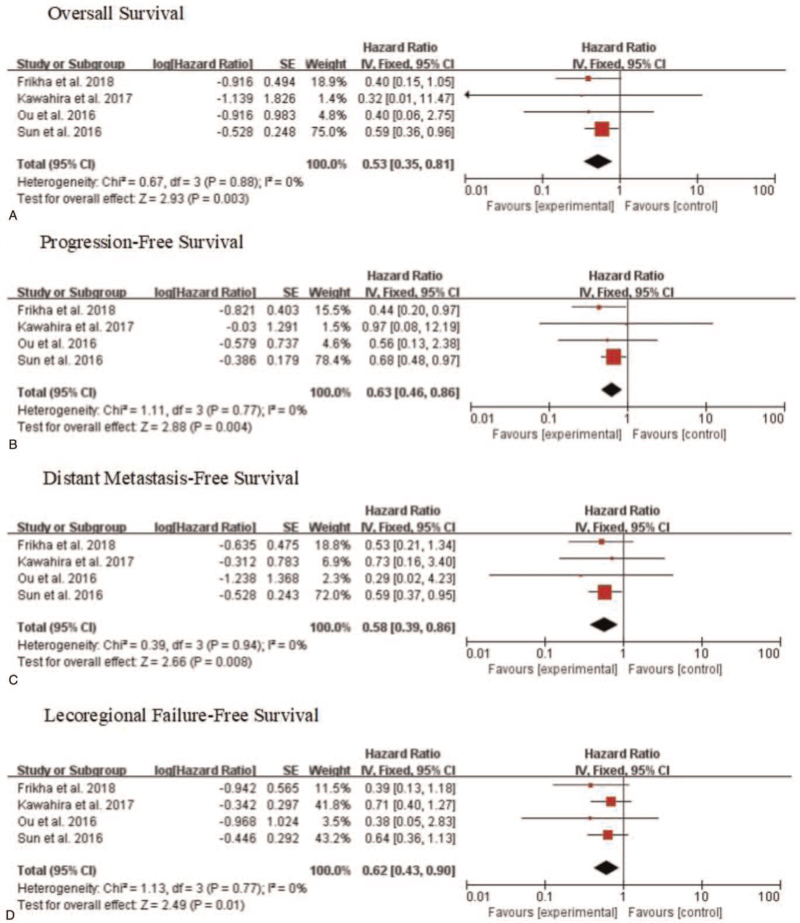
Forest plots showing the survival outcomes between TPF plus CCRT (experimental group) and CCRT (control group). (A) Overall survival; (B) Progression-free survival; (C) Distant metastasis-free survival; (D) Lecoregional failure-free survival.

OS was reported in 4 articles^[26–29]^ with a total of 697 participants. It is shown that OS was significantly improved in patients treated with TPF+ CCRT compared to those treated with CCRT alone (HR = 0.53, 95% CI: 0.35–0.81, *P* = .003, Fig. 2A). A fixed-effect model was employed because a significant difference was not obtained in the heterogeneity test (*I*^2^ = 0%, *P* = .88, Fig. 2A).

### 3.4 Progression-free survival

Four studies^[26–29]^ were eligible for analyzing PFS. No significant heterogeneity was identified (*I*^2^ = 0%, *P* = .77) and as a result, a fixed-effect model was employed to calculate pooled data. The data demonstrated that the PFS of CCRT+TPF group was significantly higher than that of CCRT group (pooled HR = 0.63, 95% CI: 0.46–0.86, *P* = .004, Fig. 2B).

### 3.5 Distant metastasis-free survival

Data about endpoints, and DMFS were extracted from 4 studies^[26–29]^ with 697 patients. No significant heterogeneity was observed among the trials (*I*^2^ = 0% *P* = .94), and therefore, a fixed-effects model was applied to synthesize the data. Compared to CCRT alone, LA-NPC patients could be more benefited from TPF-based IC plus CCRT (pooled HR = 0.58, 95% CI: 0.39–0.86, *P* = .008, Fig. 2C).

### 3.6 Loco regional failure-free survival

LRFFS data were extracted from 4 articles with 697 patients. LRFFS was significantly higher in TPF + CCRT group than that of control, with an HR of 0.62 (95% CI: 0.43–0.90, *P* = .01, Fig. 2D). The heterogeneity test showed no statistically significant difference among studies (*I*^2^ = 0%, *P* = .77), and therefore, a fixed-effects model was introduced.

### 3.7 Adverse events

Chemotherapy toxicity was reported in all recruited studies. Toxicity (grade ≥3) during treatment was evaluated according to the Common Terminology Criteria for Adverse Events (CTCAE). As shown in Table 2, TPF + CCRT was associated with an increased risk of hematological toxicities, such as leukopenia (pooled OR = 1.84, 95% CI: 0.42–8.03, *P* = .42), neutropenia (pooled OR = 1.78, 95% CI: 0.23–13.82, *P* = .58), thrombocytopenia (pooled OR = 1.76, 95% CI: 0.53–5.81, *P* = .35), and febrile neutropenia (pooled OR = 2.76, 95% CI: 0.07–101.89, *P* = .58) compared to CCRT alone.

**Table 2:** Treatment-related adverse events.

	Availability			Effect		Heterogeneity		
Adverse event (grade ≥3)	Trials (N)	TPF + CCRT (events/total)	CCRT (events/total)	OR (95% CI)	*P* value	*I* ^2^	*P* value	Analysis model
Hematological								
Leucopenia	2	103/251	49/254	1.84 (0.42–8.03)	.42	73%	.05	Random-effects
Neutropenia	3	111/283	31/284	1.78 (0.23–13.82)	.58	90%	<.0001	Random-effects
Anemia	3	9/283	9/284	1.04 (0.41–2.63)	.94	47%	.15	Fixed-effects
Thrombocytopenia	3	7/283	4/284	1.76 (0.53–5.81)	.35	0	.50	Fixed-effects
Febrile neutropenia	2	7/251	1/254	2.76 (0.07–101.89)	.58	64%	.10	Random-effects
Non-hematological								
Vomiting	3	68/283	1/284	18.94 (0.99–362.02)	.05	69%	.04	Random-effects
Dry mouth	2	16/251	13/254	2.23 (0.22–22.57)	.50	59%	.12	Random-effects
Nausea	2	51/251	46/254	0.60 (0.08–4.61)	.62	70%	.07	Random-effects
Hepatoxicity	2	7/251	3/154	1.50 (0.39–5.71)	.55	0	.39	Fixed effect
Stomatitis	3	119/283	111/284	1.15 (0.82–1.62)	.41	0	.66	Fixed-effects
Dermatitis	4	17/341	24/332	0.68 (0.36–1.30)	.24	24%	.27	Fixed-effects
Diarrhea	2	0/44	1/46	0.41 (0.02–11.05)	.60	–	–	–

For non-hematological toxicity, the TPF+CCRT regimen only significantly increased the risks of adverse events of vomiting (OR = 18.94, 95% CI: 0.99–362.02, *P* = .05) and dry mouth (OR = 2.23, 95% CI: 0.22–22.57, *P* = .50) compared with those of CCRT regimen alone. No significant difference in the incidence of other non-hematological adverse events was detected (Table 2).

## 4 Discussion

Recent evidences have proven that IC is able to reduce local failure and eradicate micro-metastasis in LA-NPC patients.^[31–33]^ However, the most optimal IC regimen for LA-NPC has not yet been determined. Therefore, we conducted a meta-analysis on exploring therapeutic efficacy and adverse events of two therapeutic options (TPF+CCRT vs CCRT) for LA-NPC, aiming to provide a basis for the selection of the final standard IC protocol. To our knowledge, this is the first comprehensive meta-analysis that directly compares therapeutic efficacy of TPF-based IC plus CCRT and CCRT alone in LA-NPC patients. Data were extracted from 2 RCTs and 3 retrospective studies from 4 countries worldwide, including 759 participants. Our results concluded that adding TPF-based IC to CCRT improves OS, PFS, DMFS, and LRFFS in LA-NPC patients.

As shown in this meta-analysis, TPF presented a pronounced efficacy on improving OS of LA-NPC. Previous clinical trials also confirmed that the TPF-based IC plus CCRT results in better survival outcomes in LA-NPC patients than other therapeutic methods.^[27,34]^ In addition, compared with the previous meta-analysis article,^[14]^ our research has added 2 high-quality retrospective studies to strengthen the accuracy of results.

We thereafter assessed PFS, and the results demonstrated that PFS of CCRT+TPF group was significantly higher than that of CCRT group, which was consistent with the results from the other 2 RCTs.^[27,29]^ However, the result differed from that of a retrospective study,^[26]^ and an insufficient sample size for the retrospective study may be the major cause. Large-scale RCTs with more participants are needed to confirm this result in the future. For the endpoints of DMFS and LRFFS, adding TPF-based IC to CCRT achieved a clear survival benefit.

Adverse events were the main causes of discontinuation of treatment plan in both experimental and control groups. Here, hematological toxicity and some non-hematological toxicities were the most frequent adverse events in LA-NPC patients. Consistent with previous studies, our results showed that TPF-based IC plus CCRT mainly increased risks of hematological toxicities, such as leukopenia, neutropenia, thrombocytopenia, febrile neutropenia. Differ from previous research, TPF regimen also increased risks of non-hematological toxicities like vomiting and dry mouth. However, these acute adverse events were uncomplicated and manageable with growth factor support, which would not affect the application of the subsequent CCRT.

There were 4 major limitations in this meta-analysis. First of all, some of the studies were non-RCTs, leading to relatively low power of our research. Secondly, drug dosage in TPF+CCRT and CCRT groups varied among studies, but it was balanced in our meta-analysis and it did not have much impact on the pooled results. Thirdly, cases of treatment-related adverse events were limited, and the significant difference may not be accurately obtained. Finally, the follow-up time varied among different studies.

## 5 Conclusions

TPF + CCRT shows a better therapeutic efficacy on LA-NPC than CCRT alone although TPF + CCRT increases the incidences of grade 3/4 acute hematological toxicity and some non-hematological toxicities.

## Author contributions

**Data curation:** Ruijuan Chen, Yongkai Lu, Yuemei Zhang, Ruixin He, Fengwen Tang.

**Formal analysis:** Ruijuan Chen, Ruixin He.

**Investigation:** Yongkai Lu, Fengwen Tang.

**Methodology:** Yongkai Lu, Wei Yuan.

**Software:** Yuemei Zhang.

**Writing – original draft:** Ruijuan Chen, Yongkai Lu, Wei Yuan, Xiaowei Zhang.

**Writing – review & editing:** Yongkai Lu, Yi Li.
